# Correction for Magnetic Field Inhomogeneities and Normalization of Voxel Values Are Needed to Better Reveal the Potential of MR Radiomic Features in Lung Cancer

**DOI:** 10.3389/fonc.2020.00043

**Published:** 2020-01-31

**Authors:** Maxime Lacroix, Frédérique Frouin, Anne-Sophie Dirand, Christophe Nioche, Fanny Orlhac, Jean-François Bernaudin, Pierre-Yves Brillet, Irène Buvat

**Affiliations:** ^1^Service d'Imagerie Médicale, AP-HP, Hôpital Avicenne, Bobigny, France; ^2^Laboratoire IMIV, UMR 1023 Inserm-CEA-Université Paris Sud, ERL 9218 CNRS, Université Paris Saclay, Orsay, France; ^3^INSERM UMR 1272 Hypoxie et Poumon, Université Paris 13, Bobigny, France

**Keywords:** lung cancer, radiomics, histological types of lung cancer, T2-weighted MR images, bias field correction, MRI normalization

## Abstract

**Purpose:** To design and validate a preprocessing procedure dedicated to T2-weighted MR images of lung cancers so as to improve the ability of radiomic features to distinguish between adenocarcinoma and other histological types.

**Materials and Methods:** A discovery set of 52 patients with advanced lung cancer who underwent T2-weighted MR imaging at 3 Tesla in a single center study from August 2017 to May 2019 was used. Findings were then validated using a validation set of 19 additional patients included from May to October 2019. Tumor type was obtained from the pathology report after trans-thoracic needle biopsy, metastatic lymph node or metastasis samples, or surgical excisions. MR images were preprocessed using N4ITK bias field correction and by normalizing voxel intensities with fat as a reference region. Segmentation and extraction of radiomic features were performed with LIFEx software on the raw images, on the N4ITK-corrected images and on the fully preprocessed images. Two analyses were conducted where radiomic features were extracted: (1) from the whole tumor volume (3D analysis); (2) from all slices encompassing the tumor (2D analysis). Receiver operating characteristic (ROC) analysis was used to identify features that could distinguish between adenocarcinoma and other histological types. Sham experiments were also designed to control the number of false positive findings.

**Results:** There were 31 (12) adenocarcinomas and 21 (7) other histological types in the discovery (validation) set. In 2D, preprocessing increased the number of discriminant radiomic features from 8 without preprocessing to 22 with preprocessing. 2D analysis yielded more features able to identify adenocarcinoma than 3D analysis (12 discriminant radiomic features after preprocessing in 3D). Preprocessing did not increase false positive findings as no discriminant features were identified in any of the sham experiments. The greatest sensitivity of the 2D analysis applied to preprocessed data was confirmed in the validation set.

**Conclusion:** Correction for magnetic field inhomogeneities and normalization of voxel values are essential to reveal the full potential of radiomic features to identify the tumor histological type from MR T2-weighted images, with classification performance similar to those reported in PET/CT and in multiphase CT in lung cancers.

## Introduction

Radiomics consists in the extraction of a large number of quantitative features from radiology images to describe the shape, intensity distribution, and texture characteristics of a region of interest (1–3). The assumption is that such image-derived features can outperform visual analysis to characterize abnormalities. In particular, in oncology, radiomic features might reflect tumor heterogeneity observed at the histological and genetic levels (4). Macroscopic structural heterogeneity can unveil differences in tumor biology, which cannot be identified by clinical data alone (5, 6). In medical images, the macroscopic heterogeneity corresponds to variations of image intensities between neighboring voxels, which are described by radiomic features. Radiomic features are thus expected to be related to the phenotype, genotype and microenvironment of the tumor, and thus be of interest to support therapeutic decisions (7). Radiomics is therefore largely investigated to assist cancer diagnosis, prognosis, and prediction of response to therapy (8, 9).

Many radiomic studies have been devoted to lung cancer, which is a major public health problem (10–16). These studies mostly focus on nodules detected on CT and/or PET scans and that can be removed surgically (8, 17, 18). CT and PET are indeed used in daily practice for managing lung cancer patients. MR images are also of interest to characterize tumors because of their excellent contrast (19–21). Yet, to the best of our knowledge, very few studies have investigated the usefulness of MR radiomic features in lung cancer patients. A study defined the optimal timing to extract radiomic features on T1-weighted images after contrast medium injection in order to predict 2-years progression-free survival (15). Another preliminary study suggested that MR-derived radiomic features (based on True Fast MR images with a Steady State Precession sequence) may improve the accuracy of models that predict the response to therapy and survivals at different time points compared to that of models based on CT features only (22). No radiomic study involving anatomical MR sequences has been reported. Predicting histology from functional MR data was reported in a meta-analysis (23) showing that diffusion MR sequences could distinguish between malignant and benign lung lesions. It was also suggested that small cell cancers had significantly lower Apparent Diffusion Coefficients than other subtypes (23). However, these functional MR sequences could not differentiate adenocarcinomas from squamous cell carcinomas.

CT intensities are expressed in Hounsfield Units (HUs) linearly related to the tissue attenuation coefficients at the energy of the CT scanner. PET images are expressed in Standardized Uptake Values (SUV) that are directly related to the tracer concentration. As a result, CT or PET image values have the same meaning, from a physics point of view, in all patient scans acquired with the same scanner and using the same protocol for the image acquisition and reconstruction. Still, the use of different scanners and/or different image acquisition and reconstruction protocols introduces some variability in voxel values hence in radiomic features (24) and some harmonization techniques have been proposed to realign radiomic features measured in different conditions (25). In anatomical MR sequences, images are initially expressed in arbitrary units, meaning that a given tissue type (for instance fat) will not always yield a similar voxel value, even when the images are acquired in the same patient and same conditions using the same scanner (26). Therefore, a measured voxel value cannot be readily interpreted in terms of well-understood physics quantity unlike in CT (a HU does correspond to a unique attenuation coefficient) and in PET (an SUV corresponds to a unique tracer concentration in a given patient). This makes radiomic studies more challenging in MR compared to PET and CT.

In that context, the present study had three objectives: (1) to design and validate a new normalization procedure dedicated to T2-weighted MR images of lung cancer patients, using subcutaneous fat as a reference tissue; (2) to perform a systematic comparison of a 3D analysis of radiomic features with a 2D analysis taking into account all slices; (3) to demonstrate the usefulness of the normalization procedure and the 2D analysis to identify relevant T2-weighted MR radiomic features for differentiating adenocarcinoma from other types of lung cancer.

MRI preprocessing, posterior to acquisitions, including magnetic bias field correction and normalization has been successfully applied to brain studies using white matter as the reference tissue (26, 27). This approach has been rarely applied to other organs. For prostate T2w images, a recent study considered muscle as the reference tissue, reporting mitigated results in terms of feature reproducibility (28). To the best of our knowledge, this normalization based on a reference tissue has never been used for lung MR images. Unlike many studies comparing 3D and 2D radiomic features where the 2D approach only exploits the slice presenting the largest tumor area or diameter, our 2D analysis calculates radiomic features in all slices and selects the median value of each feature as being representative of the tumor.

## Materials and Methods

### Population

Patient imaging, pathology, and clinical data were selected from the single-center MRI-omics database built as part of a retrospective study approved by the Institutional Review Board (protocol 32-2016, study number: 2016-A00813-48). All patients gave their written informed consents. From August 2017 to May 2019, patients with advanced lung cancer referred for brain MR imaging to detect cerebral metastases were proposed to undergo additional lung MRI sequences. Among 83 eligible patients, 8 were not included because the pathology of the tumor was not available, and 23 were excluded because of motion artifacts or incomplete MR protocol (Figure 1). The final population thus included 52 patients (34 men and 18 women) from 44 to 89 years old (mean age 66 years; SD 11.3 years). Among these patients, 42 subjects (80%) were active smokers and 2 (4%) were exposed to asbestos. These 52 patients defined the discovery set of the current study. In addition, the following 21 patients enrolled between May and October 2019 were used to create a validation set. Two were excluded because of inconsistent slice thickness. This additional population included 17 men and 2 women from 41 to 85 years old (mean age 71 years; SD 12.1 years). Among them, 13 (68%) were active smokers and 1 (5%) exposed to asbestos.

**Figure 1:**
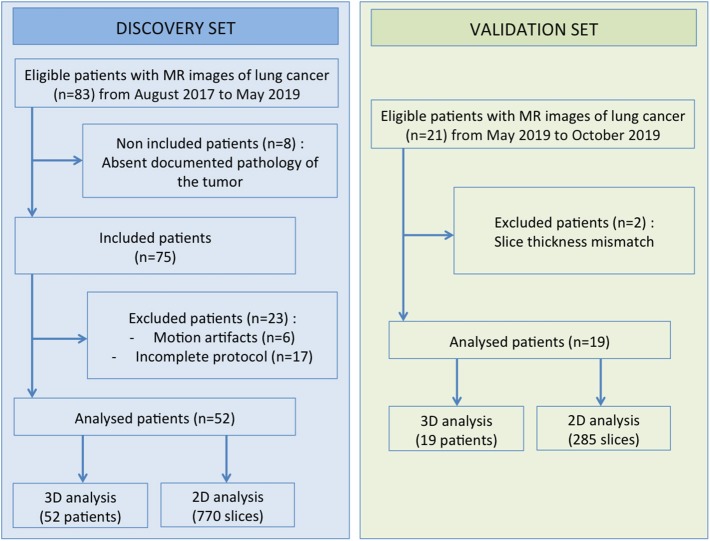
Data selection pipelines.

### MRI Acquisition

All acquisitions were performed with a 3T MRI unit (Discovery MR750, GE Healthcare, Waukesha—WI, USA), using an 18-channel phased-array body coil on the thorax. All study participants were scanned in the supine position with the arms along the body. All patients had a T2-weighted (T2w) sequence with a vendor-specific implementation of the periodically rotated overlapping parallel lines with enhanced reconstruction technique (PROPELLER) acquired in free breathing. The T2w PROPELLER sequence was selected since it provided few motion artifacts (29) and a good image quality (30, 31). The main parameters of the sequence are given in Table 1.

**Table 1:** Parameters of MR images acquisition protocols.

**Parameter**	**T2w PROPELLER**
Plane	Axial
TR (ms)	9,677
TE (ms)	96
FA (degree)	160
FOV (mm)	500 × 500
Matrix	240 × 240
Slice thickness (mm)	4
Inter slice spacing (mm)	0
Frequence	384
NEx	1.5
Gating	Respiratory
Breath hold	No
Acquisition time (s)	65

*TR, repetition time; TE, echo time; FA, flip angle; FOV, field of view; NEx, number of excitations*.

### Pathological Assessments of Tumor Samples

A dedicated pathologist (JB, with more than 30 years of experience in lung cancer pathology), blinded to the MR findings, reviewed all pathology reports and filled a structured pathology worksheet. The pathology information came from trans-thoracic core needle biopsy (*n* = 31 in the discovery set, *n* = 11 in the validation set), metastatic lymph node samples (*n* = 11 in the discovery set, *n* = 3 in the validation set), other metastatic location samples (*n* = 6 in the discovery set, *n* = 4 in the validation set), and surgical excisions (*n* = 4 in the discovery set, *n* = 1 in the validation set). Detailed characteristics are reported in Table 2.

**Table 2:** Tumor characteristics.

**Population**	**Discovery set**	**Validation set**
**Number of cases**	52	19
**Type of tumor**: ***n*** **(%)**		
Adenocarcinoma	31 (60%)	12 (63%)
Other types	21 (40%)	7 (37%)
Squamous cell carcinoma	16 (76%)	4 (57%)
Small cell carcinoma	2 (9.5%)	1 (14%)
Sarcomatoid tumor	2 (9.5%)	2 (29%)
Large cell carcinoma	1 (5%)	0
**Mean size in long axis (mm)**	63.4 ± 23.2	67.7 ± 21.1
	(Range: 23–110)	(Range: 27–109)
**Location:** ***n*** **(%)**		
Right upper lobe	24 (46%)	10 (53%)
Middle lobe	3 (6%)	0
Right lower lobe	8 (15%)	4 (21%)
Left upper lobe	10 (19%)	3 (16%)
Left lower lobe	7 (14%)	2 (10%)
**T status (Lung-cancer TNM 8th edition):** ***n*** **(%)**		
T1	3 (6%)	1 (5%)
T2	4 (8%)	2 (11%)
T3	10 (19%)	5 (26%)
T4	35 (67%)	11 (58%)
**Invasion**		
No parietal or mediastinal invasion	17 (33%)	6 (32%)
Parietal invasion	18 (35%)	4 (21%)
Mediastinal invasion	13 (25%)	8 (42%)
Parietal and mediastinal invasion	4 (7%)	1 (5%)

Based on the pathology worksheet, two groups were defined: the first group included all patients with adenocarcinoma and the second group included all other patients.

### MR Image Analysis

Lung MR images were retrieved from the Pictorial Archive and Communication System (Carestream 3.2. Carestream Health, Rochester, New York), anonymized and loaded in a workstation for radiomic analysis. Preprocessing of images included two steps: a correction for magnetic field (B1) inhomogeneity in order to reduce the signal intensity variation across the field of view, followed by a normalization of intensities based on the delineation of a reference tissue. Tumors were then segmented and 3D and 2D radiomic features were extracted for raw data, N4ITK corrected data, and normalized N4ITK-corrected data. Statistical analyses were performed to identify the features that could distinguish between the group of adenocarcinoma and the group including other tumor types.

#### Correction of Magnetic Field Inhomogeneity

Magnetic field inhomogeneity artifacts were corrected based on the estimation of a bias field constrained to be spatially smooth (32). The bias field was estimated with the publicly available N4ITK algorithm using ANTs software (http://stnava.github.io/ANTs) with the standard setting of hyper-parameters. Each voxel value in the raw image was then modified by dividing its value by the corresponding voxel value in the bias field. This approach is widely used for brain studies (26), but not for other organs. It reduces variations of the mean intensity between similar tissues located at different positions within the field of view.

#### Image Intensity Normalization

Significant variations in mean intensity values measured in similar tissues (for instance subcutaneous fat for lung studies, white matter for brain studies) can be observed between different patients even when using a similar acquisition protocol on the same scanner (26). These variations are a major pitfall for radiomic studies (26). The intensity normalization aims at reducing the intensity variations between different patients. The proposed approach relies on the definition of a reference region that is always in the field of view of thoracic acquisitions, namely the fat. When compared to vertebra and muscle, fat was chosen as the most appropriate reference region because it showed the smallest intra-patient variability (see Results section). Three 2D regions-of-interest (ROIs) were therefore drawn in the normal subcutaneous fat while avoiding vessels, with each region drawn in a different slice. A linear transform was then applied to every image voxel v so that the mean value of the reference tissue was equal to 0 and its standard deviation was equal to 1:

$$\begin{array}{l} \text{Is}\left(\text{v}\right)=\left[\text{I}\left(\text{v}\right)-\text{F}\right]/\sigma , \end{array}$$

where I(v) is the original intensity of each voxel in the bias field corrected image, F and σ are the mean intensity and associated standard deviation over all voxels belonging to the three fat regions, and Is(v) is the intensity in voxel v of the normalized image.

#### Segmentation

An expert radiologist with 4 years experience in thoracic imaging segmented the tumors using the LIFEx software (www.lifexsoft.org) (33). A coarse region surrounding the tumor was manually defined and then refined using an intensity threshold manually set for each patient to delineate the tumor from the lung tissue. The borders between the tumor, the mediastinum and the chest wall were manually delineated. The tumor volume was defined as a single 3D connected component. To further investigate the impact of the segmentation on the results, the original ROIs were modified by automatically shrinking the contours by two pixels.

#### Radiomic Feature Extraction

Radiomic features were computed in the tumor region for both the raw, the N4ITK-corrected and the normalized N4ITK-corrected MR images using the LIFEx software compliant with the Image Biomarker Standardization Initiative guidelines (https://arxiv.org/abs/1612.07003). Features included shape features, first-order features that do not account for the spatial arrangement of voxel values, and second-order (textural) features that reflect how voxel values are spatially arranged. The definition of the matrices needed for textural feature calculations requires gray level quantization. For raw images, N4ITK corrected images, and normalized N4ITK images, fixed bin sizes were used for that gray level quantization step. The bin size was chosen so that 256 bins always encompassed all voxel values observed in the tumors, yielding a bin size of 15 units for raw images, 10 units for N4ITK corrected images, and 0.2 for normalized N4ITK corrected images.

For each patient and each image (without and with preprocessing), two sets of features were extracted. A first set of 48 3D radiomic features was obtained from the 3D tumor volume. All feature names are given in Supplemental Tables 2, 3 and precisely defined in the LIFEx online documentation (www.lifexsoft.org). A second set of 46 2D radiomic features was extracted for each slice of the tumor, by performing the calculations in the largest 2D connected component present in the slice (the two 3D shape features were not calculated in 2D analysis). Slices with too small regions (<64 pixels) were removed from the analysis, as calculating second-order features in regions with <64 pixels could be meaningless (34). Using the 2D approach, the median value of each feature among the whole set of slices encompassing the patient tumor was defined as the representative value of the corresponding feature for that tumor. Therefore, for each approach, called 2D and 3D in the following, each patient was associated with one 2D value and one 3D value for each feature. Our 2D approach was compared to the conventional 2D approach that consists in selecting the feature value measured from the slice including the largest tumor area.

### Classification Tasks

To test the predictive power of each radiomic feature, we determined the ability of each feature to distinguish between adenocarcinoma (ADK) and other tumors (OTH). This task is further referred to as the ADK task. To check the relevance of our findings, a “sham” task was also used by randomly defining a sham ADK group and a sham OTH group. To do so, each patient was randomly assigned to the sham ADK or sham OTH group, whatever the actual tumor type of the patient, but still using the same prevalence of ADK as in the real data (31 patients in the sham ADK group and 21 patients in the sham OTH group). This task is further referred as the RAND task.

### Statistical Analysis

To assess the impact of the correction for magnetic field inhomogeneity (N4ITK correction) on voxel values throughout the image volume, three ROIs were manually drawn in the vertebra body of Th3, Th4, and Th5, three ROIs were drawn in the pectoral muscles, in addition to the three ROIs defined in the fat (Figure 2). For each type of tissue, the coefficient of variation defined as the standard deviation divided by the mean over all the voxels belonging to the different ROIs of the same tissue was calculated in the original images and in the images after the N4ITK correction. Paired Wilcoxon signed-rank tests were used to determine whether the N4ITK correction significantly impacted the coefficients of variation.

**Figure 2:**
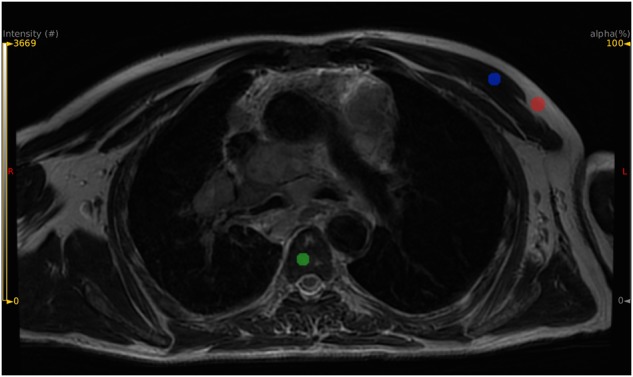
Example of ROI positioning for three candidate reference tissues: subcutaneous fat in red color, vertebral body in green color, pectoral muscle in blue color.

To investigate the ability of radiomic feature to predict whether the tumor was an ADK or an OTH tumor, ROC analysis was performed for each feature, and resulting areas under the curve (AUC) were computed. 2D and 3D feature values were used, as calculated from the raw images, from the images after N4ITK correction and from the fully preprocessed images, i.e., normalized N4ITK corrected images. Following (35), the *p*-value of the Wilcoxon sum rank test was used to test whether the AUC differed significantly from 0.5. Features for which *p*-value was <0.05 were thus selected as candidate discriminant features. The same ROC analyses were performed for the RAND task. To reduce the possible false discovery rate, features that remained significant after Benjamini-Hochberg correction for multiple tests were also identified. All these analyses were performed separately using the discovery set and the validation set.

## Results

### Pathological Data

The pathologic characteristics of tumors are listed in Table 2. In the discovery set, there were 31 adenocarcinomas (ADK group) and 21 other histological types (OTH group). The OTH group contained a majority of squamous cell carcinoma (76%). In the validation set, there were 12 adenocarcinomas and 7 other histological types, with a majority of squamous cell carcinoma (57%).

### Impact of the Correction of Magnetic Field Inhomogeneity on Voxel Values

Figure 3 shows an example of a bias field as estimated by the N4ITK algorithm for one patient. As expected, the largest variations are observed between the center of the field of view and the periphery near the coil. For the three types of tissue (fat, vertebra, and pectoral muscle), the coefficients of variation demonstrated a statistically significant reduction after bias field correction (see Supplemental Table 1). As fat yielded the smallest coefficient of variation, it was chosen as the reference tissue.

**Figure 3:**
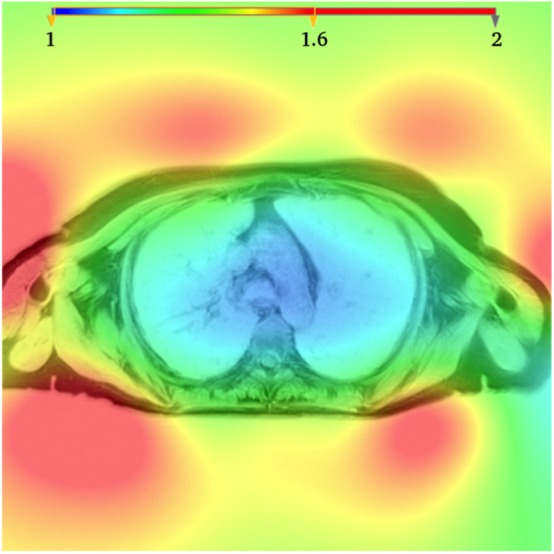
Bias field as estimated using the N4ITK algorithm. The bias field is displayed in color and superimposed to the image in gray scale.

### Segmentation

Figure 4 shows two examples of tumor segmentation with LIFEx, highlighting the signal heterogeneity within these tumors.

**Figure 4:**
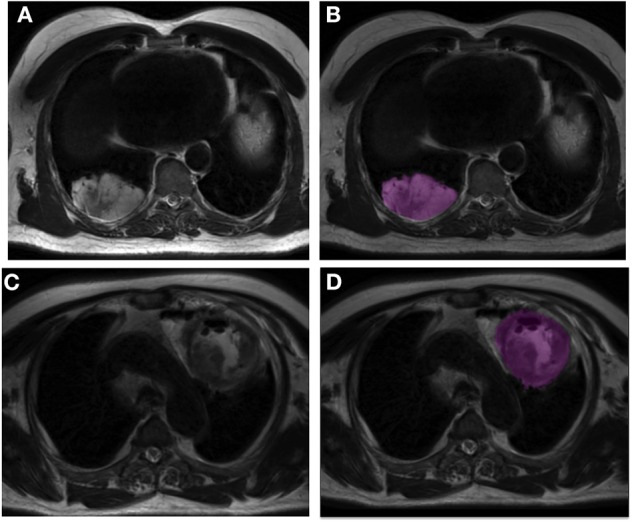
Example of tumor segmentation for two patients. First row: patient with a lung adenocarcinoma of the right lower lobe (long axis: 77 mm). Raw image **(A)** and image after N4ITK correction with the segmented tumor volume in pink **(B)**. Second row: patient with a squamous cell carcinoma of the left upper lobe (long axis: 93 mm). Raw image **(C)** and image after N4ITK correction with the segmented tumor volume in pink **(D)**.

### Impact of Pre-processing on the Predictive Values of Radiomic Features

The discovery set was first analyzed. Table 3 summarizes the significant features (AUC significantly greater that 0.5, *p* < 0.05) when using the 2D and 3D approaches, without preprocessing, with N4ITK correction only and with preprocessing involving the two steps (N4ITK correction and normalization), for both real data and sham data. Supplemental Tables 2, 3 provide the AUC and associated 95% confidence intervals for each feature. For the ADK classification task, 8 discriminant features are extracted systematically whatever the configuration tested (except GLRLM_GLNU for 3D N4ITK corrected data, and HISTO_Skewness for 2D N4ITK corrected data, both having a *p*-value of 0.054). These 8 features (HISTO_Skewness, SHAPE_Volume, GLCM_Correlation, GLRLM_GLNU, GLRLM_RLNU, NGLDM_Coarseness, GLZLM_GLNU, GLZLM_ZLNU) are subsequently called common discriminant features. For the RAND task, no feature yielded an AUC significantly different from 0.5. Correcting for the magnetic field inhomogeneity did not substantially change the number of predictive features. However, when combining bias field correction and normalization, some additional predictive features were observed especially for the 2D configuration with 14 new discriminant features in addition to the 8 common discriminant features. In the 3D analysis, the feature yielding the largest AUC was the “GLCM_Correlation” textural feature with an AUC of 0.77. The same feature yielded the largest AUC in the 2D analysis, with an AUC of 0.82. Figure 5 shows the associated boxplot corresponding to the 2D analysis for the ADK and OTH groups.

**Table 3:** Number and list of features with an AUC significantly >0.5 for the different analyses (3D and 2D for raw data, N4ITK corrected data, and N4ITK corrected and normalized data—ADK task based on real data and RAND task based on sham data).

	**Raw data**	**N4ITK corrected data**	**N4ITK corrected and normalized data**	
**3D FEATURES (DISCOVERY SET)**				
ADK task	8 (**1**)	7 (**1**)	12 (**1**)	
RAND task	0	0	0	
Feature name	HISTO_Skewness SHAPE_Volume **GLCM_Correlation** GLRLM_GLNU GLRLM_RLNU NGLDM_Coarseness GLZLM_GLNU GLZLM_ZLNU	HISTO_Skewness SHAPE_Volume **GLCM_Correlation** GLRLM_RLNU NGLDM_Coarseness GLZLM_GLNU GLZLM_ZLNU	HISTO_Skewness SHAPE_Volume **GLCM_Correlation** GLRLM_LRE GLRLM_GLNU GLRLM_RLNU NGLDM_Coarseness NGLDM_Busyness GLZLM_SZE GLZLM_LZE GLZLM_GLNU GLZLM_ZLNU	
**2D FEATURES (DISCOVERY SET)**				
ADK task	8 (**5**)	9 (**4**)	22 (**20**)	
RAND task	0	0	0	
Feature name	HISTO_Skewness **SHAPE_Volume** **GLCM_Correlation** GLRLM_GLNU **GLRLM_RLNU** NGLDM_Coarseness **GLZLM_GLNU** **GLZLM_ZLNU**	**SHAPE_Volume** **GLCM_Correlation** GLCM_Entropy_log2 GLCM_Entropy_log10 GLRLM_GLNU **GLRLM_RLNU** NGLDM_Coarseness GLZLM_GLNU **GLZLM_ZLNU**	HISTO_Skewness **SHAPE_Volume** **GLCM_Homogeneity** **GLCM_Contrast** **GLCM_Correlation** **GLCM_Entropy_log2** **GLCM_Entropy_log10** **GLCM_Dissimilarity** **GLRLM_SRE** **GLRLM_LRE** **GLRLM_GLNU**	**GLRLM_RLNU GLRLM_RP NGLDM_Coarseness** **NGLDM_Contrast** **NGLDM_Busyness** **GLZLM_SZE** **GLZLM_LZE** GLZLM_SZHGE **GLZLM_GLNU** **GLZLM_ZLNU** **GLZLM_ZP**

*Bold numbers in brackets give the numbers of significant features after Benjamini-Hochberg correction for multiple comparisons, with corresponding feature names in bold*.

**Figure 5:**
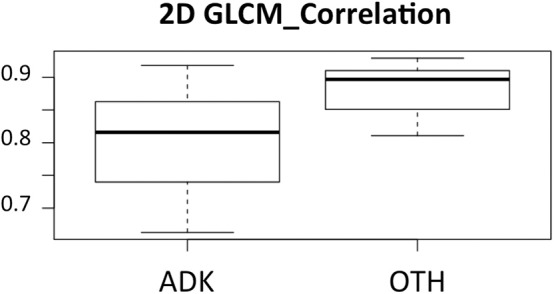
Boxplot showing the values of the 2D “GLCM-correlation” feature for the group of patients with adenocarcinomas (ADK) and the group of patients having a different histological status (OTH).

The validation set was analyzed with the same approach. Table 4 shows the significantly predictive features for all configurations. Compared with the discovery set, less significantly predictive features were identified, partly due to the lower number of patients hence larger confidence intervals (see examples in Supplemental Table 4). In all cases, the significant features were part of the 8 common discriminant features identified in the discovery set.

**Table 4:** Number and list of features with an AUC significantly >0.5 in the validation set for the 3D and 2D analyses (raw data, N4ITK corrected data, and N4ITK corrected and normalized data—ADK task based on real data).

	**Raw data**	**N4ITK corrected data**	**N4ITK corrected and normalized data**
**3D FEATURES (VALIDATION SET)**			
ADK task	0	1	1
Feature name		GLCM_Correlation	GLCM_Correlation
**2D FEATURES (VALIDATION SET)**			
ADK task	4	3	6
Feature name	SHAPE_Volume GLRLM_GLNU NGLDM_Coarseness GLZLM_GLNU	SHAPE_Volume GLRLM_RLNU NGLDM_Coarseness	SHAPE_Volume GLRLM_GLNU GLRLM_RLNU NGLDM_Coarseness GLZLM_GLNU GLZLM_ZLNU

In the best configuration (2D analysis), a Wilcoxon signed-rank test showed that there was no statistically significant difference between the AUC of the eight common discriminant features for the different tested configurations: raw data vs. N4ITK corrected data, raw data vs. normalized N4ITK corrected data, and N4ITK corrected data vs. normalized N4ITK corrected data, and this was true for both the discovery and the validation sets (Table 5). There were statistically significant differences between the AUC of the 14 additional features (revealed on the discovery set) for the normalized N4ITK corrected data when compared to raw data or N4ITK corrected data (Table 5) and again, this was observed both for the discovery set and for the validation set.

**Table 5:** Paired Wilcoxon signed rank tests to compare AUC between (1) raw data and N4ITK corrected data, (2) raw data and N4ITK corrected and normalized data, (3) N4ITK corrected data and N4ITK corrected and normalized data for the discriminant features (common and additional) using the discovery and the validation sets.

	**2D analysis**			
**AUC comparison**	**Common discriminant features** **(*****n*** **=** **8)**		**Additional discriminant features** **(*****n*** **=** **14)**	
	**Discovery set**	**Validation set**	**Discovery set**	**Validation set**
N4ITK corrected data vs. raw data	ns	ns	ns	ns
N4ITK corrected and normalized data vs. raw data	ns	ns	*p* = 0.001^**^	*p* = 0.001^**^
N4ITK corrected and normalized data vs. N4ITK corrected data	ns	ns	*p* = 0.001^**^	*p* = 0.003^**^

*ns, not significant*.

** *Stands for p-values smaller than 0.005*.

### Impact of Segmentation and of 2D Analysis on the Predictive Values of Radiomic Features

Supplemental Table 5 demonstrates the low impact of the tumor border erosion on the 2D discriminant features. Indeed eight features (the eight common discriminant features) were revealed using both the initial tumor regions and the eroded regions in the raw data, nine in the N4ITK corrected data (including seven of the eight common discriminant features), and 22 in the N4ITK corrected and normalized data (the eight common discriminant features and the 14 additional features shown in Table 3).

Supplemental Table 6 shows the interest of using our 2D approach, selecting the median value of the 2D features computed for all slices encompassing the tumor as opposed to the conventional 2D approach that calculates the feature value from the slice including the largest tumor area. Indeed the number of discriminant features was always superior with our 2D approach except for one supplemental feature for the raw data. For instance we found 6 additional discriminant features for N4ITK corrected and normalized data using our 2D approach instead of the conventional 2D approach.

## Discussion

In this study, we investigated the potential of MRI radiomics for lung cancer assessment and demonstrated the need for careful preprocessing of MR images to identify radiomic features correlated with the tumor pathology. While CT and PET scans are the standard imaging procedures to manage lung cancer patients, the clinical workflow can easily include additional lung acquisitions when MRI is prescribed for brain metastasis screening. Here, we focused on an anatomical T2w PROPELLER sequence that produced good quality images in the lung area as assessed by the radiologists in our department. We determined whether radiomic features calculated from these T2-weighted images could predict whether the tumor was an ADK, a question that has already been addressed using CT or PET radiomics (10, 36–40). The best reported performance from non-injected CT scans was an AUC of 0.72 (multivariate analysis) to differentiate ADK and squamous cell carcinomas (36). Using enhanced CT, an AUC of 0.86 was reported at the venous phase for the same classification task (10). Radiomic features extracted from PET/CT could also differentiate adenocarcinoma from other histological types (37–40) with an AUC of 0.81 reported in Kirienko et al. (38), and a radiomic signature to distinguish ADK from squamous cell carcinoma with an AUC of 0.90 reported in Zhu et al. (40).

Interestingly, we found that several MR radiomic features analyzed independently yielded an AUC >0.65 and up to 0.82. Yet, identifying these features required thorough preprocessing, without which up to 66% of the informative features (14 out of the 22 in the 2D approach, see Supplemental Table 3) were not identified as such.

The need for some preprocessing steps before extracting MRI radiomic features has been very recently acknowledged for other tumor types (41, 42). Although there is no consensus on the preprocessing methods that should be used, two main pitfalls that are specific to MRI have been identified. The first one results from the B1 magnetic field inhomogeneities (43, 44) caused by MR gradients that introduces variability in signal intensity of a given tissue type as a function of its location within the field of view. This bias is more severe in high field MR and was present in our 3T data. The second challenge is the significant variation in pixel values between different patients (42, 45, 46) in anatomical MR images, even when using the same scanner and the same acquisition sequences, due to the arbitrary units used to represent the anatomical MR images.

In our work, we proposed two complementary approaches to deal with these two issues. A bias field correction was performed using the N4ITK method, which is the state-of-art method for brain studies. N4ITK is a histogram based technique that estimates a slowly varying bias field by maximizing the high frequency histogram content in the image (32). Our goal was to validate its use in thoracic imaging and assess its impact on subsequent radiomic analysis. Using normal tissues, such as the vertebral bodies, subcutaneous fat, and pectoral muscle, we demonstrated that this correction was successful at reducing the variations of voxel values in all these tissues (Supplemental Table 1), the largest effect being observed in the subcutaneous fat. This is very likely due to the fat peripheral location. The identification of radiomic features able to predict ADK tumors was only slightly impacted by this correction (Table 3). Indeed this correction did not aim at increasing the identification of informative radiomic features, but at improving the subsequent normalization procedure, by reducing the coefficients of variation in the reference tissue. Other techniques of bias field correction, such as B1 mapping could also be of interest. Yet, a definite advantage of N4ITK is that it can be retrospectively used, which is especially useful as many radiomic studies are still performed retrospectively.

The challenge of image intensity normalization was addressed by defining a reference tissue. We chose the subcutaneous fat as it was always present in the thoracic field of view and showed the lowest coefficient of variation within patients (Supplemental Table 1). The principle of the normalization was to arbitrarily set the MR intensity to 0 in fat regions and its associated standard deviation to 1, similar to setting Hounsfield Units to 0 in water in CT imaging. Doing so, for any patient, the value will be 0 in the fat for these anatomical T2-weighted PROPELLER images, and all image values will be scaled linearly. The linear transformation is a very simple model with respect to the complexity of the MR signal intensity and more sophisticated models could certainly be used, but our aim was to determine whether this simple transformation could already reduce the variability of MR signal intensity across patients hence increase the statistical power of MR radiomic analysis. Our results suggest that the number of informative features for identifying ADK is substantially increased when using the image intensity normalization combined with the bias field correction (Table 3). In the 3D approach, four additional features were identified while in the 2D analysis, 14 additional features were identified as discriminant. The eight common discriminant features that were already identified as informative before preprocessing remained informative after preprocessing, demonstrating that these features were robust with respect to the magnetic field heterogeneity and intensity scaling. Indeed five (SHAPE_Volume, GLRLM_GLNU, GLRLM_RLNU, GLZLM_GLNU, GLZLM_ZLNU) of these eight features are highly correlated with the volume of the region of interest (34), and that volume remains identical whatever the preprocessing steps. The question of whether the 14 new radiomic features identified as informative after preprocessing were truly informative for the classification task or were “false positive” features was answered by designing the sham experiment and by analyzing the validation set. In the sham experiment, we knew that we should not find any feature that would be related to the “fake” ADK or OTH status of the tumors, as each tumor was randomly assigned as ADK or OTH, whatever its actual pathological report. Table 3 confirmed that without preprocessing, with N4ITK correction, and with full preprocessing, no feature was identified as informative of the fake tumor type. Table 5 shows that the trends observed on the discovery set for the 14 additional features identified using the normalized N4ITK corrected data were confirmed on the validation set. All additional features were textural features, demonstrating the need for preprocessing to compute robust discriminant textural features. Altogether, these results demonstrate that the preprocessing does not produce an inflation of false positive and suggests that the additional features identified in the real classification task are truly informative.

In our cohort, all images had the same voxel size. It was thus not necessary to resample the images as previously proposed (47, 48) to reduce the variability induced by different voxel size. To characterize the tumor type based on the MR radiomic features, we compared two approaches: a 2D approach where radiomic features were computed in each slice and the median value over all slices was chosen as the representative value for the tumor, and a 3D approach in which the features directly pertain to the whole tumor volume. The 2D approach identified more informative radiomic features than then 3D approach (Table 3). Several hypotheses might explain this result. First, voxels are not isotropic, because the slice thickness (4 mm) is greater than the intra-plane voxel size (0.8 mm). As a result, 3D calculation of second-order feature is biased. Another reason might be the large size of most tumors in our study. All patients had advanced tumors with a mean diameter of 63 ± 23 mm, so each slice already contained a representative view of the tumor that might be sufficient to estimate the tumor type (see Figure 4). Two previous studies compared 2D and 3D radiomic feature performance for lung cancer in CT (49, 50). The first one did not find any significant difference between 2D and 3D results (49), while the second study reported better performance using the 3D analysis (50). Yet, for these two studies, the 2D analysis was limited to the slice that included the largest cross-section of the lesion, while in our so-called 2D approach, we still accounted for all slices encompassing the tumor. The selection of one single slice might lead to information loss while our 2D approach used all 2D slices to end up with a single feature value per tumor volume. Our 2D approach identified more discriminant features than the one-slice based 2D approach (Supplemental Table 6), especially for N4ITK corrected and normalized data.

The feature that yielded the largest AUC was GLCM_Correlation. This feature has actually already been reported as predictive in other MRI radiomic study: lower values of GLCM_Correlation on Diffusion Weighted Images and higher values of GLCM_Contrast on T2w sequences were shown to be correlated to an early disease progression in rectal cancers (51).

There are several limitations in our study. First, our results related to the prediction of ADK should be confirmed on a larger cohort. As all data were acquired in the same institution and using the same scanner, our findings would also need a multi-center validation. Another limitation is due to the fact that only one operator segmented the tumors and the robustness of the findings with respect to the tumor delineation should be further investigated (52). To investigate the impact of the tumor delineation on our results, all the segmented tumors were automatically eroded by an element of size 1.5 mm and results were similar, confirming the greater sensitivity of 2D analysis on normalized N4ITK corrected data to identify discriminant features (see Supplemental Table 5). This suggests that for tumors with large volumes as in our study, significant variations in results due to small changes in tumor contour delineation are unlikely. Histology was mostly determined by trans-thoracic core needle biopsy, which might not be representative of the whole tumor volume. This is a definite limitation as lung tumors may have heterogeneous histological types depending on the location in the lesion (53). Our task was to distinguish between ADK and all other tumor types, so this second tumor group was quite heterogeneous in itself. The reason why we did not separate the OTH group into different tumor types was to keep enough tumors in each group for the classification task. Last, the best prediction accuracy we obtained (AUC of 0.82) is not sufficient for clinical applicability (36). This accuracy might be limited by the fact that we used univariate models only, because of the relatively small size of our cohort. Our results warrant multivariate analyses based on larger patient cohorts. Also, we focused on one MR sequence only, while combining radiomic features from different MR sequences might be useful to enhance the accuracy of the classification.

## Conclusion

We demonstrated that MRI T2-weighted sequences of lung cancer patients yielded radiomic features related to the pathological tumor type and that the number of informative radiomic features was significantly increased by appropriate processing of the MR images. Key preprocessing steps are correction for the magnetic field inhomogeneity and normalization of the voxel values to set a intensity scale common to all patient images. In addition, in our cohort, the 2D analysis selecting the median value of each feature among the different slices encompassing the tumor volume revealed more discriminant radiomic features than the 3D analysis. Based on these results, further exploration of the potential of MR radiomics in lung cancer patients is warranted.

## Data Availability Statement

The datasets for this article are not publicly available because they were extracted from an on-going clinical study (IRB protocol 32-2016, study number: 2016-A00813-48). Requests to access the datasets should be directed to Pierre-Yves Brillet, pierre-yves.brillet@aphp.fr.

## Ethics Statement

The studies involving human participants were reviewed and approved by Comité de Protection des Personnes d'Ile de France Paris X. The patients/participants provided their written informed consent to participate in this study.

## Author Contributions

ML, FF, P-YB, and IB contributed to the conception and design of the study. ML and P-YB collected the image and clinical data. J-FB did the pathological records reviewing. ML and FF performed the image analysis. CN and FO developed some specific software components. A-SD and IB performed the statistical analysis. ML wrote the first draft of the manuscript. FF and IB wrote some sections of the manuscript. All authors contributed to manuscript revision, read, and approved the submitted version.

### Conflict of Interest

The authors declare that the research was conducted in the absence of any commercial or financial relationships that could be construed as a potential conflict of interest.
