# Supplementary material for: Correction for Magnetic Field Inhomogeneities and Normalization of Voxel Values Are Needed to Better Reveal the Potential of MR Radiomic Features in Lung Cancer
Source: Front Oncol. 2020 Jan 31;10:43. doi: 10.3389/fonc.2020.00043 (PMC7006432; doi:10.3389/fonc.2020.00043)
Supplement: Supplementary file 1 [file Data_Sheet_1.PDF]

Supplemental table 1: Mean ± standard deviation of intra-patient coefficient of variation (CV) in fat, vertebra, and muscle (see Figure 2 for an example of ROI positioning). For each tissue, three ROIS have been draw and coefficients of variation were computed in the fusion of the three ROIs. Paired Wilcoxon signed rank tests show that CV of N4ITK corrected data are statistically smaller than CV of raw data, (p-values in brackets).

CV (%)	Fat	Vertebra	Muscle
Raw data	8.7±4.0	18.7± 7.7	32.3±12.9
N4ITK corrected data	6.9±3.7 ($p<10^{-5}$)	18.2±7.7 ($p=0.02$)	31.6±12.6 ($p<10^{-4}$)
