# Supplementary material for: Correction for Magnetic Field Inhomogeneities and Normalization of Voxel Values Are Needed to Better Reveal the Potential of MR Radiomic Features in Lung Cancer
Source: Front Oncol. 2020 Jan 31;10:43. doi: 10.3389/fonc.2020.00043 (PMC7006432; doi:10.3389/fonc.2020.00043)
Supplement: Supplementary file 2 [file Data_Sheet_2.pdf]

Supplemental Table 2: 3D analysis results. AUC of ROC curves (and associated 95% confidence intervals) are shown before preprocessing (raw data), after N4ITK correction and after full preprocessing (N4ITK + normalization) for the real and sham data. AUC values have been tested (Wilcoxon rank-sum test) to determine values significantly different from 0.5 (p-value less than 0.05, 0.005 and 0.0005 denoted as *, **, and *** respectively). P-value less than 0.05 after Benjamini-Hochberg correction for multiple comparisons are highlighted in green. The 8 common discriminative features are in blue color.

**Feature name**†	**3D raw data (true)**		**3D N4ITK corrected data (true)**		**3D N4ITK corrected and normalized data (true)**		**3D raw data (sham)**		**3D N4ITK corrected data (sham)**		**3D N4ITK corrected and normalized data (sham)**	
CONVENTIONAL_#min	0.63	(0.47-0.79)	0.64	(0.48-0.8)	0.52	(0.36-0.68)	0.54	(0.37-0.71)	0.57	(0.4-0.73)	0.50	(0.34-0.67)
CONVENTIONAL_#mean	0.53	(0.36-0.69)	0.52	(0.35-0.69)	0.51	(0.34-0.67)	0.45	(0.27-0.62)	0.48	(0.31-0.65)	0.51	(0.35-0.67)
CONVENTIONAL_#std	0.63	(0.48-0.78)	0.61	(0.46-0.77)	0.60	(0.44-0.76)	0.55	(0.39-0.71)	0.45	(0.29-0.61)	0.52	(0.36-0.69)
CONVENTIONAL_#max	0.59	(0.43-0.74)	0.43	(0.27-0.59)	0.63	(0.47-0.78)	0.55	(0.38-0.71)	0.56	(0.4-0.72)	0.52	(0.36-0.68)
CONVENTIONAL_#Q1	0.51	(0.35-0.68)	0.55	(0.38-0.73)	0.50	(0.34-0.67)	0.45	(0.27-0.63)	0.48	(0.31-0.64)	0.51	(0.35-0.67)
CONVENTIONAL_#Q2	0.49	(0.33-0.66)	0.47	(0.3-0.64)	0.51	(0.35-0.68)	0.45	(0.27-0.62)	0.48	(0.31-0.65)	0.50	(0.34-0.66)
CONVENTIONAL_#Q3	0.53	(0.37-0.69)	0.49	(0.32-0.65)	0.51	(0.34-0.67)	0.46	(0.29-0.63)	0.49	(0.32-0.66)	0.56	(0.4-0.72)
HISTO_Skewness	0.72*	(0.57-0.87)	0.67*	(0.52-0.83)	0.67*	(0.52-0.83)	0.53	(0.37-0.69)	0.54	(0.38-0.71)	0.55	(0.38-0.71)
HISTO_Kurtosis	0.66	(0.51-0.81)	0.64	(0.48-0.79)	0.64	(0.48-0.79)	0.59	(0.43-0.74)	0.60	(0.44-0.76)	0.60	(0.44-0.76)
HISTO_ExcessKurtosis	0.66	(0.51-0.81)	0.64	(0.48-0.79)	0.64	(0.48-0.79)	0.59	(0.43-0.74)	0.60	(0.44-0.76)	0.60	(0.44-0.76)
HISTO_Entropy_log10	0.63	(0.47-0.78)	0.60	(0.45-0.76)	0.54	(0.38-0.7)	0.45	(0.28-0.61)	0.45	(0.29-0.61)	0.52	(0.36-0.69)
HISTO_Entropy_log2	0.63	(0.47-0.78)	0.60	(0.45-0.76)	0.54	(0.38-0.7)	0.45	(0.28-0.61)	0.45	(0.29-0.61)	0.52	(0.36-0.69)
HISTO_Energy	0.62	(0.46-0.78)	0.60	(0.45-0.75)	0.53	(0.37-0.7)	0.46	(0.3-0.62)	0.46	(0.3-0.62)	0.55	(0.38-0.71)
SHAPE_Volume (mL)	0.71*	(0.57-0.85)	0.71*	(0.57-0.85)	0.71*	(0.57-0.85)	0.49	(0.33-0.65)	0.49	(0.33-0.65)	0.49	(0.33-0.65)
SHAPE_Sphericity	0.64	(0.49-0.8)	0.64	(0.49-0.8)	0.64	(0.49-0.8)	0.50	(0.34-0.66)	0.50	(0.34-0.66)	0.50	(0.34-0.66)
SHAPE_Compacity	0.64	(0.49-0.79)	0.64	(0.49-0.79)	0.64	(0.49-0.79)	0.53	(0.37-0.69)	0.53	(0.37-0.69)	0.53	(0.37-0.69)
GLCM_Homogeneity	0.54	(0.38-0.7)	0.48	(0.32-0.65)	0.64	(0.49-0.8)	0.55	(0.38-0.71)	0.05	(0.33-0.66)	0.51	(0.34-0.67)
GLCM_Energy	0.58	(0.43-0.74)	0.59	(0.44-0.75)	0.51	(0.35-0.67)	0.56	(0.4-0.72)	0.55	(0.38-0.71)	0.55	(0.39-0.72)
GLCM_Contrast	0.52	(0.36-0.68)	0.52	(0.35-0.68)	0.62	(0.47-0.78)	0.54	(0.38-0.71)	0.50	(0.33-0.66)	0.51	(0.35-0.68)
GLCM_Correlation	0.77**	(0.64-0.91)	0.77**	(0.64-0.91)	0.77**	(0.63-0.91)	0.54	(0.38-0.7)	0.54	(0.38-0.7)	0.54	(0.37-0.7)
GLCM_Entropy_log10	0.62	(0.47-0.78)	0.62	(0.47-0.78)	0.46	(0.3-0.62)	0.56	(0.4-0.72)	0.57	(0.41-0.73)	0.59	(0.43-0.75)
GLCM_Entropy_log2	0.62	(0.47-0.78)	0.62	(0.47-0.77)	0.46	(0.3-0.62)	0.56	(0.4-0.72)	0.57	(0.41-0.73)	0.59	(0.43-0.75)

GLCM_Dissimilarity	0.51	(0.35-0.67)	0.51	(0.34-0.67)	0.62	(0.47-0.78)	0.53	(0.37-0.7)	0.49	(0.32-0.65)	0.51	(0.35-0.68)
GLRLM_SRE	0.59	(0.43-0.75)	0.55	(0.39-0.71)	0.66	(0.51-0.81)	0.53	(0.36-0.69)	0.51	(0.35-0.68)	0.51	(0.34-0.67)
GLRLM_LRE	0.59	(0.43-0.75)	0.56	(0.4-0.72)	0.67*	(0.52-0.82)	0.53	(0.36-0.7)	0.51	(0.34-0.67)	0.50	(0.34-0.67)
GLRLM_LGRE	0.53	(0.37-0.7)	0.57	(0.4-0.74)	0.48	(0.31-0.64)	0.45	(0.28-0.63)	0.50	(0.33-0.66)	0.51	(0.35-0.67)
GLRLM_HGRE	0.53	(0.37-0.7)	0.52	(0.35-0.69)	0.61	(0.45-0.77)	0.45	(0.28-0.63)	0.48	(0.31-0.65)	0.49	(0.32-0.65)
GLRLM_SRLGE	0.53	(0.36-0.69)	0.57	(0.4-0.74)	0.47	(0.31-0.63)	0.46	(0.28-0.63)	0.50	(0.34-0.67)	0.51	(0.35-0.68)
GLRLM_SRHGE	0.53	(0.37-0.7)	0.51	(0.34-0.68)	0.61	(0.45-0.77)	0.45	(0.28-0.63)	0.48	(0.31-0.65)	0.48	(0.31-0.65)
GLRLM_LRLGE	0.45	(0.28-0.61)	0.59	(0.42-0.76)	0.51	(0.35-0.67)	0.47	(0.3-0.64)	0.52	(0.35-0.69)	0.52	(0.36-0.68)
GLRLM_LRHGE	0.53	(0.36-0.69)	0.52	(0.35-0.69)	0.59	(0.43-0.75)	0.45	(0.27-0.62)	0.48	(0.31-0.65)	0.48	(0.31-0.64)
GLRLM_GLNU	0.68*	(0.54-0.83)	0.66	(0.51-0.81)	0.69*	(0.54-0.83)	0.47	(0.31-0.63)	0.54	(0.38-0.7)	0.47	(0.31-0.63)
GLRLM_RLNU	0.70*	(0.56-0.84)	0.71*	(0.57-0.85)	0.70*	(0.56-0.84)	0.53	(0.37-0.69)	0.53	(0.37-0.69)	0.48	(0.32-0.64)
GLRLM_RP	0.59	(0.43-0.75)	0.56	(0.4-0.72)	0.66	(0.51-0.81)	0.53	(0.36-0.69)	0.51	(0.34-0.67)	0.51	(0.34-0.67)
NGLDM_Coarseness	0.69*	(0.54-0.83)	0.69*	(0.55-0.84)	0.69*	(0.55-0.84)	0.53	(0.37-0.69)	0.53	(0.37-0.69)	0.53	(0.37-0.69)
NGLDM_Contrast	0.60	(0.44-0.76)	0.55	(0.39-0.71)	0.62	(0.47-0.78)	0.54	(0.38-0.71)	0.55	(0.39-0.72)	0.45	(0.28-0.61)
NGLDM_Busyness	0.65	(0.49-0.82)	0.63	(0.47-0.79)	0.69*	(0.54-0.83)	0.51	(0.35-0.67)	0.51	(0.35-0.67)	0.50	(0.34-0.66)
GLZLM_SZE	0.58	(0.41-0.74)	0.57	(0.4-0.73)	0.71*	(0.57-0.85)	0.54	(0.37-0.7)	0.50	(0.34-0.66)	0.53	(0.37-0.69)
GLZLM_LZE	0.61	(0.45-0.77)	0.56	(0.4-0.72)	0.67*	(0.51-0.82)	0.53	(0.37-0.7)	0.51	(0.35-0.68)	0.51	(0.34-0.67)
GLZLM_LGZE	0.50	(0.33-0.66)	0.54	(0.37-0.71)	0.58	(0.42-0.74)	0.45	(0.28-0.63)	0.51	(0.34-0.68)	0.51	(0.35-0.67)
GLZLM_HGZE	0.54	(0.38-0.7)	0.51	(0.34-0.68)	0.60	(0.44-0.76)	0.45	(0.28-0.63)	0.48	(0.31-0.65)	0.49	(0.32-0.66)
GLZLM_SZLGE	0.52	(0.36-0.68)	0.51	(0.34-0.68)	0.63	(0.47-0.78)	0.46	(0.29-0.64)	0.50	(0.33-0.67)	0.50	(0.34-0.66)
GLZLM_SZHGE	0.53	(0.37-0.7)	0.52	(0.35-0.69)	0.63	(0.48-0.79)	0.45	(0.28-0.63)	0.48	(0.31-0.64)	0.47	(0.3-0.64)
GLZLM_LZLGE	0.62	(0.46-0.78)	0.61	(0.45-0.77)	0.65	(0.49-0.8)	0.53	(0.36-0.7)	0.54	(0.38-0.71)	0.51	(0.34-0.67)
GLZLM_LZHGE	0.61	(0.45-0.76)	0.57	(0.42-0.73)	0.63	(0.47-0.78)	0.49	(0.33-0.65)	0.52	(0.36-0.68)	0.48	(0.32-0.65)
GLZLM_GLNU	0.70*	(0.56-0.85)	0.69*	(0.54-0.83)	0.71*	(0.56-0.85)	0.52	(0.36-0.68)	0.53	(0.37-0.69)	0.52	(0.36-0.68)
GLZLM_ZLNU	0.72*	(0.58-0.86)	0.73*	(0.59-0.86)	0.67*	(0.53-0.82)	0.52	(0.36-0.68)	0.48	(0.32-0.64)	0.50	(0.34-0.66)
GLZLM_ZP	0.58	(0.42-0.74)	0.55	(0.39-0.71)	0.66	(0.51-0.81)	0.52	(0.35-0.69)	0.51	(0.34-0.67)	0.51	(0.34-0.67)

[†]A full description of features is available on the LIFEx website:
www.lifexsoft.org/images/phocagallery/documentation/ProtocolTexture/UserGuide/TextureUserGuide.pdf

Supplemental Table 3: 2D analysis results. AUC of ROC curves (and associated 95% confidence intervals) are shown before preprocessing (raw data), after N4ITK correction and after full preprocessing (N4ITK + normalization) for the real and the sham data. AUC values have been tested (Wilcoxon rank-sum test) to determine values significantly different from 0.5 (p-value less than 0.05, 0.005 and 0.0005 denoted as *, **, and *** respectively). P-value less than 0.05 after Benjamini-Hochberg correction for multiple comparisons are highlighted in green. The 8 common discriminative features are in blue color.

**Feature name**	**2D raw data (true)**		**2D N4ITK corrected data (true)**		**2D N4ITK corrected and normalized data (true)**		**2D raw data (sham)**		**2D N4ITK corrected data (sham)**		**2D N4ITK corrected and normalized data (sham)**	
CONVENTIONAL_#min	0.58	(0.42-0.74)	0.56	(0.39-0.73)	0.52	(0.36-0.68)	0.54	(0.36-0.71)	0.59	(0.43-0.75)	0.50	(0.34-0.66)
CONVENTIONAL_#mean	0.52	(0.35-0.68)	0.52	(0.35-0.69)	0.51	(0.35-0.67)	0.45	(0.27-0.62)	0.48	(0.31-0.65)	0.52	(0.36-0.68)
CONVENTIONAL_#std	0.63	(0.48-0.79)	0.62	(0.47-0.78)	0.60	(0.44-0.76)	0.44	(0.28-0.61)	0.45	(0.29-0.62)	0.53	(0.37-0.69)
CONVENTIONAL_#max	0.61	(0.45-0.76)	0.58	(0.42-0.74)	0.63	(0.47-0.79)	0.57	(0.4-0.74)	0.58	(0.42-0.74)	0.49	(0.33-0.65)
CONVENTIONAL_#Q1	0.52	(0.36-0.69)	0.55	(0.38-0.72)	0.51	(0.35-0.67)	0.44	(0.27-0.62)	0.48	(0.31-0.65)	0.52	(0.36-0.68)
CONVENTIONAL_#Q2	0.50	(0.34-0.67)	0.53	(0.36-0.7)	0.52	(0.36-0.68)	0.45	(0.27-0.62)	0.47	(0.31-0.64)	0.51	(0.35-0.67)
CONVENTIONAL_#Q3	0.53	(0.36-0.69)	0.49	(0.32-0.66)	0.51	(0.34-0.67)	0.47	(0.29-0.64)	0.49	(0.32-0.66)	0.54	(0.38-0.7)
HISTO_Skewness	0.68*	(0.53-0.83)	0.66	(0.5-0.82)	0.66*	(0.51-0.82)	0.51	(0.35-0.68)	0.49	(0.33-0.65)	0.49	(0.33-0.65)
HISTO_Kurtosis	0.59	(0.43-0.75)	0.55	(0.39-0.71)	0.55	(0.39-0.71)	0.61	(0.45-0.78)	0.63	(0.48-0.79)	0.63	(0.48-0.79)
HISTO_ExcessKurtosis	0.59	(0.43-0.75)	0.55	(0.39-0.71)	0.55	(0.39-0.71)	0.61	(0.45-0.78)	0.63	(0.48-0.79)	0.63	(0.48-0.79)
HISTO_Entropy_log10	0.62	(0.47-0.78)	0.64	(0.49-0.79)	0.50	(0.34-0.67)	0.44	(0.28-0.61)	0.55	(0.38-0.71)	0.47	(0.3-0.65)
HISTO_Entropy_log2	0.62	(0.47-0.78)	0.64	(0.49-0.79)	0.50	(0.34-0.67)	0.44	(0.28-0.61)	0.55	(0.38-0.71)	0.47	(0.3-0.65)
HISTO_Energy	0.61	(0.46-0.77)	0.63	(0.47-0.78)	0.52	(0.36-0.68)	0.54	(0.37-0.7)	0.47	(0.31-0.63)	0.54	(0.37-0.72)
SHAPE_Volume (mL)	0.76**	(0.63-0.89)	0.76**	(0.63-0.89)	0.76**	(0.63-0.89)	0.52	(0.36-0.68)	0.52	(0.36-0.68)	0.52	(0.36-0.68)
GLCM_Homogeneity	0.57	(0.41-0.73)	0.55	(0.38-0.71)	0.73**	(0.59-0.87)	0.57	(0.4-0.74)	0.47	(0.3-0.63)	0.59	(0.43-0.74)
GLCM_Energy	0.61	(0.45-0.76)	0.64	(0.49-0.79)	0.64	(0.48-0.79)	0.56	(0.39-0.72)	0.55	(0.39-0.71)	0.55	(0.4-0.71)
GLCM_Contrast	0.55	(0.39-0.71)	0.53	(0.36-0.69)	0.76**	(0.63-0.89)	0.57	(0.4-0.74)	0.53	(0.37-0.7)	0.59	(0.43-0.75)
GLCM_Correlation	0.83***	(0.72-0.94)	0.82***	(0.71-0.93)	0.82***	(0.71-0.93)	0.47	(0.31-0.64)	0.49	(0.32-0.65)	0.48	(0.32-0.65)
GLCM_Entropy_log10	0.64	(0.48-0.79)	0.67*	(0.52-0.81)	0.71*	(0.57-0.85)	0.57	(0.41-0.73)	0.56	(0.4-0.72)	0.46	(0.3-0.62)
GLCM_Entropy_log2	0.64	(0.48-0.79)	0.67*	(0.52-0.81)	0.71*	(0.57-0.85)	0.57	(0.41-0.73)	0.56	(0.4-0.72)	0.46	(0.3-0.62)
GLCM_Dissimilarity	0.54	(0.38-0.7)	0.52	(0.36-0.69)	0.73**	(0.59-0.87)	0.57	(0.4-0.74)	0.47	(0.31-0.64)	0.59	(0.43-0.74)

GLRLM_SRE	0.58	(0.42-0.74)	0.56	(0.4-0.72)	0.73**	(0.59-0.87)	0.56	(0.39-0.73)	0.48	(0.32-0.64)	0.57	(0.42-0.73)
GLRLM_LRE	0.59	(0.43-0.75)	0.57	(0.41-0.73)	0.74**	(0.6-0.88)	0.56	(0.39-0.73)	0.49	(0.32-0.65)	0.58	(0.42-0.74)
GLRLM_LGRE	0.53	(0.37-0.7)	0.54	(0.37-0.72)	0.56	(0.4-0.72)	0.43	(0.26-0.61)	0.48	(0.31-0.65)	0.55	(0.39-0.71)
GLRLM_HGRE	0.53	(0.37-0.69)	0.49	(0.32-0.66)	0.64	(0.48-0.79)	0.46	(0.28-0.63)	0.48	(0.31-0.65)	0.50	(0.33-0.67)
GLRLM_SRLGE	0.52	(0.36-0.69)	0.54	(0.37-0.72)	0.57	(0.41-0.73)	0.44	(0.26-0.61)	0.48	(0.31-0.65)	0.44	(0.28-0.6)
GLRLM_SRHGE	0.53	(0.37-0.7)	0.5	(0.33-0.67)	0.66	(0.51-0.81)	0.46	(0.29-0.64)	0.52	(0.35-0.69)	0.50	(0.33-0.67)
GLRLM_LRLGE	0.47	(0.31-0.64)	0.55	(0.38-0.73)	0.55	(0.38-0.71)	0.45	(0.27-0.63)	0.49	(0.32-0.66)	0.57	(0.41-0.72)
GLRLM_LRHGE	0.52	(0.36-0.69)	0.49	(0.33-0.66)	0.61	(0.45-0.76)	0.44	(0.27-0.62)	0.48	(0.31-0.65)	0.53	(0.37-0.7)
GLRLM_GLNU	0.72*	(0.58-0.86)	0.67*	(0.53-0.82)	0.74**	(0.6-0.87)	0.53	(0.37-0.69)	0.54	(0.38-0.7)	0.55	(0.39-0.71)
GLRLM_RLNU	0.75**	(0.62-0.89)	0.76**	(0.63-0.89)	0.75**	(0.62-0.88)	0.53	(0.37-0.69)	0.54	(0.38-0.7)	0.53	(0.37-0.69)
GLRLM_RP	0.58	(0.42-0.74)	0.57	(0.41-0.73)	0.73**	(0.59-0.88)	0.56	(0.39-0.73)	0.49	(0.32-0.65)	0.58	(0.42-0.74)
NGLDM_Coarseness	0.71*	(0.57-0.85)	0.71*	(0.57-0.85)	0.69*	(0.55-0.83)	0.56	(0.4-0.72)	0.55	(0.39-0.71)	0.57	(0.41-0.72)
NGLDM_Contrast	0.62	(0.46-0.77)	0.57	(0.41-0.74)	0.71*	(0.57-0.85)	0.55	(0.39-0.71)	0.56	(0.4-0.72)	0.63	(0.47-0.78)
NGLDM_Busyness	0.53	(0.36-0.7)	0.53	(0.36-0.7)	0.71*	(0.57-0.85)	0.55	(0.38-0.71)	0.53	(0.37-0.69)	0.51	(0.35-0.68)
GLZLM_SZE	0.56	(0.39-0.72)	0.56	(0.39-0.72)	0.75**	(0.61-0.89)	0.57	(0.4-0.74)	0.47	(0.31-0.63)	0.55	(0.4-0.71)
GLZLM_LZE	0.58	(0.42-0.75)	0.57	(0.41-0.73)	0.75**	(0.61-0.88)	0.56	(0.39-0.73)	0.49	(0.32-0.65)	0.58	(0.43-0.74)
GLZLM_LGZE	0.53	(0.36-0.69)	0.55	(0.37-0.72)	0.59	(0.43-0.75)	0.44	(0.27-0.61)	0.48	(0.31-0.65)	0.55	(0.39-0.71)
GLZLM_HGZE	0.54	(0.38-0.71)	0.51	(0.34-0.68)	0.65	(0.49-0.8)	0.45	(0.28-0.63)	0.52	(0.35-0.69)	0.51	(0.34-0.67)
GLZLM_SZLGE	0.50	(0.34-0.67)	0.53	(0.36-0.71)	0.63	(0.47-0.78)	0.44	(0.27-0.62)	0.48	(0.31-0.65)	0.55	(0.39-0.71)
GLZLM_SZHGE	0.54	(0.37-0.7)	0.51	(0.34-0.68)	0.67*	(0.52-0.82)	0.45	(0.28-0.63)	0.52	(0.35-0.69)	0.52	(0.35-0.69)
GLZLM_LZLGE	0.55	(0.38-0.71)	0.57	(0.4-0.74)	0.53	(0.37-0.7)	0.56	(0.38-0.74)	0.51	(0.35-0.68)	0.56	(0.4-0.73)
GLZLM_LZHGE	0.57	(0.41-0.73)	0.53	(0.37-0.7)	0.51	(0.35-0.67)	0.55	(0.38-0.72)	0.55	(0.38-0.72)	0.55	(0.39-0.71)
GLZLM_GLNU	0.73**	(0.59-0.87)	0.69*	(0.54-0.83)	0.74**	(0.6-0.87)	0.55	(0.39-0.71)	0.55	(0.39-0.71)	0.55	(0.39-0.71)
GLZLM_ZLNU	0.74**	(0.6-0.87)	0.77**	(0.64-0.9)	0.73**	(0.6-0.87)	0.54	(0.38-0.7)	0.57	(0.41-0.73)	0.53	(0.38-0.69)
GLZLM_ZP	0.57	(0.41-0.74)	0.55	(0.39-0.72)	0.74**	(0.6-0.88)	0.57	(0.4-0.74)	0.48	(0.32-0.65)	0.57	(0.41-0.73)

Supplemental Table 4: 2D analysis results in the validation set (n=19 patients) for the features identified as discriminant in the discovery set. The 8 common discriminative features revealed on the discovery set are in blue color. AUC of ROC curves (and associated 95% confidence intervals) are shown before preprocessing (raw data), after N4ITK correction and after full preprocessing (N4ITK + normalization). AUC values significantly different from 0.5 (p-value < 0.05) are denoted as *).

**Feature name**	**2D raw data (true)**	**2D N4ITK corrected data (true)**	**2D N4ITK corrected and normalized data (true)**
HISTO_Skewness	0.58 (0.3-0.86)	0.60 (0.3-0.89)	0.60 (0.3-0.89)
SHAPE_Volume (mL)	0.80* (0.58-1)	0.80* (0.58-1)	0.80* (0.58-1)
GLCM_Homogeneity	0.58 (0.24-0.93)	0.56 (0.27-0.85)	0.67 (0.4-0.93)
GLCM_Contrast	0.56 (0.2-0.92)	0.57 (0.28-0.87)	0.63 (0.36-0.9)
GLCM_Correlation	0.69 (0.42-0.96)	0.67 (0.4-0.94)	0.68 (0.41-0.95)
GLCM_Entropy_log10	0.50 (0.14-0.86)	0.67 (0.37-0.97)	0.71 (0.46-0.97)
GLCM_Entropy_log2	0.50 (0.14-0.86)	0.67 (0.37-0.97)	0.71 (0.46-0.97)
GLCM_Dissimilarity	0.58 (0.21-0.95)	0.55 (0.25-0.84)	0.64 (0.38-0.91)
GLRLM_SRE	0.62 (0.29-0.94)	0.52 (0.23-0.82)	0.67 (0.4-0.93)
GLRLM_LRE	0.61 (0.28-0.94)	0.52 (0.23-0.82)	0.68 (0.42-0.94)
GLRLM_GLNU	0.80* (0.57-1)	0.68 (0.42-0.94)	0.82* (0.61-1)
GLRLM_RLNU	0.76 (0.52-1)	0.83* (0.61-1)	0.85* (0.66-1)
GLRLM_RP	0.62 (0.29-0.94)	0.52 (0.23-0.82)	0.68 (0.42-0.94)
NGLDM_Coarseness	0.82* (0.62-1)	0.82* (0.62-1)	0.81* (0.61-1)
NGLDM_Contrast	0.67 (0.38-0.95)	0.58 (0.31-0.86)	0.67 (0.41-0.92)
NGLDM_Busyness	0.63 (0.32-0.95)	0.48 (0.19-0.76)	0.75 (0.52-0.98)
GLZLM_SZE	0.62 (0.29-0.95)	0.51 (0.22-0.81)	0.65 (0.39-0.92)
GLZLM_LZE	0.62 (0.29-0.94)	0.54 (0.24-0.83)	0.69 (0.44-0.94)
GLZLM_SZHGE	0.45 (0.13-0.77)	0.64 (0.35-0.94)	0.57 (0.28-0.86)
GLZLM_GLNU	0.81* (0.61-1)	0.77 (0.55-1)	0.86* (0.68-1)
GLZLM_ZLNU	0.71 (0.44-0.99)	0.75 (0.47-1)	0.86* (0.68-1)
GLZLM_ZP	0.61 (0.28-0.94)	0.51 (0.22-0.81)	0.65 (0.39-0.92)
