# Supplementary material for: Correction for Magnetic Field Inhomogeneities and Normalization of Voxel Values Are Needed to Better Reveal the Potential of MR Radiomic Features in Lung Cancer
Source: Front Oncol. 2020 Jan 31;10:43. doi: 10.3389/fonc.2020.00043 (PMC7006432; doi:10.3389/fonc.2020.00043)
Supplement: Supplementary file 3 [file Data_Sheet_3.PDF]

Supplemental Table 5: Number of features with an AUC significantly greater than 0.5 using original ROIs and eroded ROIs. The last row gives the features found significant both using the original ROIs and the eroded ROIs. Bold numbers in brackets give the numbers of significant features after Benjamini-Hochberg correction for multiple comparisons, with corresponding feature names are in bold.

Number of significant 2D features (Discovery set)			
	Raw data	N4ITK corrected data	N4ITK corrected and normalized data
Initial ROIs	8 (**5**)	9 (**4**)	22 (**20**)
ROIs after 1.5 mm erosion	11 (**4**)	10 (**4**)	26 (**22**)
Features found in both cases	HISTO_Skewness<br>**SHAPE_Volume**<br>**GLCM_Correlation**<br>GLRLM_GLNU<br>**GLRLM_RLNU**<br>NGLDM_Coarseness<br>GLZLM_GLNU<br>**GLZLM_ZLNU**	**SHAPE_Volume**<br>**GLCM_Correlation**<br>GLCM_Entropy_log2<br>GLCM_Entropy_log10<br>GLRLM_GLNU<br>**GLRLM_RLNU**<br>NGLDM_Coarseness<br>GLZLM_GLNU<br>**GLZLM_ZLNU**	**HISTO_Skewness**<br>**SHAPE_Volume**<br>GLCM_Homogeneity<br>**GLCM_Contrast**<br>**GLCM_Correlation**<br>**GLCM_Entropy_log2**<br>**GLCM_Entropy_log10**<br>**GLCM_Dissimilarity**<br>**GLRLM_SRE**<br>GLRLM_LRE<br>**GLRLM_GLNU**<br>**GLRLM_RLNU**<br>**GLRLM_RP**<br>**NGLDM_Coarseness**<br>**NGLDM_Contrast**<br>**NGLDM_Busyness**<br>**GLZLM_SZE**<br>GLZLM_LZE<br>**GLZLM_SZHGE**<br>**GLZLM_GLNU**<br>**GLZLM_ZLNU**<br>**GLZLM_ZP**

Supplemental Table 6: Number of features with an AUC significantly greater than 0.5 using our 2D approach, selecting the median value of the 2D features computed for all slices encompassing the tumor and the conventional approach 2D approach, selecting the feature value in the slice including the largest tumor area. Numbers in brackets give the numbers of significant features after Benjamini-Hochberg correction for multiple comparisons, with corresponding feature names in bold.

Number of significant 2D features (Discovery set)			
	Raw data	N4ITK corrected data	N4ITK corrected and normalized data
Our global 2D approach	8 (**5**)	9 (**4**)	22 (**20**)
One-slice based 2D approach	9 (**1**)	5 (**1**)	16 (**12**)
Features found in both cases	HISTO_Skewness<br>SHAPE_Volume<br>**GLCM_Correlation**<br>GLRLM_GLNU<br>GLRLM_RLNU<br>GLZLM_GLNU<br>GLZLM_ZLNU	SHAPE_Volume<br>**GLCM_Correlation**<br>GLRLM_RLNU<br>GLZLM_ZLNU	**SHAPE_Volume**<br>**GLCM_Homogeneity**<br>**GLCM_Contrast**<br>**GLCM_Correlation**<br>**GLCM_Dissimilarity**<br>**GLRLM_SRE**<br>**GLRLM_LRE**<br>GLRLM_GLNU<br>**GLRLM_RLNU**<br>**GLRLM_RP**<br>NGLDM_Contrast<br>**GLZLM_SZE**<br>**GLZLM_LZE**<br>GLZLM_GLNU<br>GLZLM_ZLNU<br>**GLZLM_ZP**
